# Theoretical Prediction of Dual-Potency Anti-Tumor Agents: Combination of Oxoplatin with Other FDA-Approved Oncology Drugs

**DOI:** 10.3390/ijms21134741

**Published:** 2020-07-03

**Authors:** José Pedro Cerón-Carrasco

**Affiliations:** Reconocimiento y Encapsulación Molecular, Universidad Católica San Antonio de Murcia Campus los Jerónimos, 30107 Murcia, Spain; jpceron@ucam.edu

**Keywords:** cancer, drug design, organometallics, platinum-based drugs, bifunctional compounds, theoretical tools

## Abstract

Although Pt(II)-based drugs are widely used to treat cancer, very few molecules have been approved for routine use in chemotherapy due to their side-effects on healthy tissues. A new approach to reducing the toxicity of these drugs is generating a prodrug by increasing the oxidation state of the metallic center to Pt(IV), a less reactive form that is only activated once it enters a cell. We used theoretical tools to combine the parent Pt(IV) prodrug, oxoplatin, with the most recent FDA-approved anti-cancer drug set published by the National Institute of Health (NIH). The only prerequisite imposed for the latter was the presence of one carboxylic group in the structure, a chemical feature that ensures a link to the coordination sphere via a simple esterification procedure. Our calculations led to a series of bifunctional prodrugs ranked according to their relative stabilities and activation profiles. Of all the designed molecules, the combination of oxoplatin with aminolevulinic acid as the bioactive ligand emerged as the most promising strategy by which to design enhanced dual-potency oncology drugs.

## 1. Introduction

**Figure 1:**
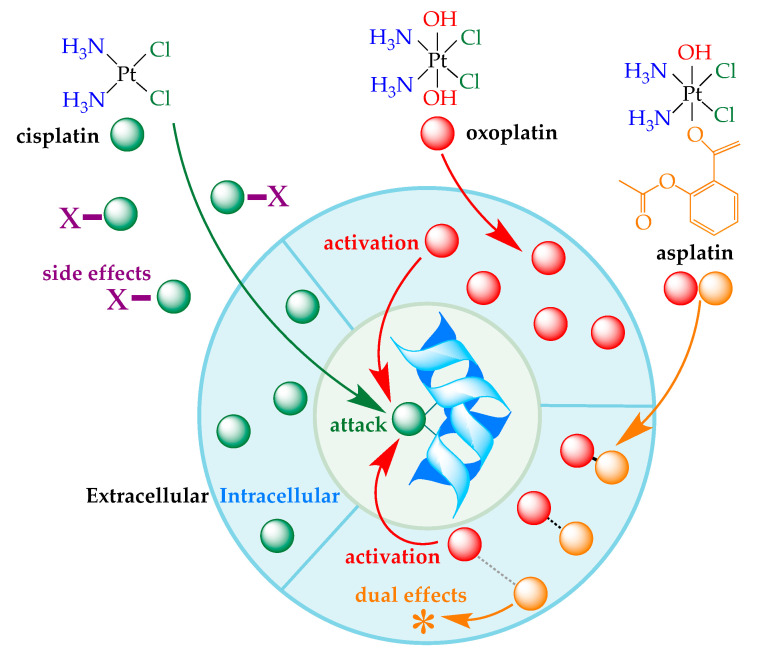
Chemical structures of the parent Pt(II)-based drug (cisplatin) and the Pt(IV) prodrugs (oxoplatin and asplatin). Schematic representation of the mechanism by which a metallodrug damages DNA. Cisplatin (shown in green) is very reactive, and the final step of the reaction produces a large distortion in the double helix structure of the DNA. However, it also reacts with the biomolecules present in the extracellular media. These non-specific interactions are the source of the main side-effects of this drug (X marked in purple). Oxoplatin (shown in red) is less reactive and enters the cell directly without any other reactions. It is activated in the intracellular medium by ascorbic acid, which reduces the Pt(IV) centers to Pt(II), leading to the in situ generation of cisplatin. In asplatin (shown as red–orange balls), one of the axial −OH ligands is replaced by aspirin. It is also activated to cisplatin inside the cell by the action of ascorbic acid. The free form of aspirin (orange ball) produces a dual-potency anti-tumor effect (denoted as an asterisk).

The unexpected discovery of the bioactivity of Pt salts by Rosenberg about 60 years ago opened the door to a new type of cancer treatment: chemotherapy with transition metals [1]. Unfortunately, only three anti-cancer drugs are routinely used in hospitals—the original cisplatin and two derivatives, carboplatin and oxoplatin [2]. Figure 1 shows that the final step in the action of cisplatin-like drugs is an attack on the cell’s DNA, which eventually blocks cell replication by disrupting the natural double helix architecture of the DNA molecule [3]. The cisplatin derivatives were not specifically designed to target cancer cells, and they react with a wide spectrum of biomolecules present in the extracellular media, which is the source of the undesirable side-effects associated with this type of chemotherapy [4]. With the aim of minimizing the risks of chemotherapy related to the high reactivity of the classical Pt(II) salts, Sadler and co-workers proposed increasing the oxidation state of the Pt to the less reactive Pt(IV) by inserting additional axial ligands into the parent cisplatin structure [5]. Figure 1 shows how the parent Pt(IV) prodrug, oxoplatin, is less toxic than the cisplatin-like drugs because it does not react in the extracellular region. Simultaneously, oxoplatin presents valuable pharmacokinetics and can be self-activated by redox reactions once it has reached the intracellular medium. The driving force for oxoplatin activation is based on the natural gradient of the concentration of ascorbic acid in the human body, which is higher inside than outside the cell, rather than on an exogenous chemical or physical agent [6]. Accordingly, the prodrug becomes reactive by only forming cisplatin within the intracellular medium.

Inspired by Sadler’s work [7], two different groups proposed the injection of a bifunctional prodrug by covering the Pt(IV) center with an active rather than a passive ligand, e.g., aspirin. In this synthetic strategy, aspirin replaces one of the axial −OH ligands, leading to asplatin, also called platin-A (Figure 1) [8,9]. The subsequent release of cisplatin upon reduction leads to the highly reactive Pt(II) agent and the free form of the bioactive ligand. The former attacks the DNA of the malignant cell while the latter reduces the mechanism of cancer growth and/or helps to mitigate the side-effects of the drug. Because they contain two active moieties (metallic center + bioactive ligand), asplatin-like molecules can be designed to produce a dual-potency effect. We used computational methods to predict the effects of replacing aspirin with other bioactive ligands. This work is therefore a further step towards designing enhanced bifunctional prodrugs.

## 2. Results

**Figure 2:**
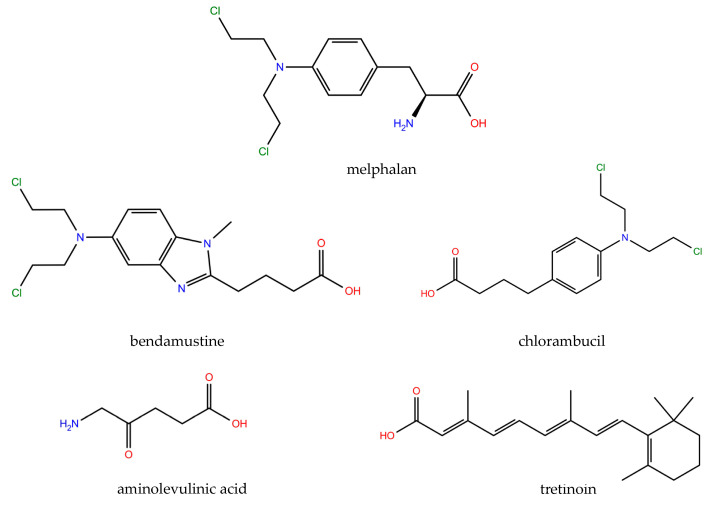
Five of the molecules in the list of approved oncology drugs included in the AOD9 set of the National Cancer Institute have one carboxylic group: melphalan, bendamustine, chlorambucil, aminolevulinic acid and tretinoin (retinoic acid). All the structures are shown as deposited in the PubChem Substance and Compound database (see text).

With the aim of proposing molecules applicable to the treatment of cancer rather than conducting a gedankenexperiment, we adapted the computational strategy of Ponte et al. [10] to screen the latest Food and Drug Administration (FDA)-approved oncology drug set (AOD9) published by the National Cancer Institute, part of the US National Institutes of Health (NIH). This set contains the most recent 147 approved anti-cancer drugs [11]. We imposed only one prerequisite: the presence of one carboxylic group in the structure that ensures a link to the Pt coordination sphere via a simple esterification reaction [12]. This systematic selection yielded five drugs: melphalan, bendamustine, chlorambucil, aminolevulinic acid and tretinoin (retinoic acid). Figure 2 shows the structures of these drugs as deposited in the PubChem database [13,14].

The successful design of asplatin suggests an effective synthetic strategy for coordinating a bioactive ligand to oxoplatin by forming a carboxylate bridge at one of the hydroxyl groups. This is, however, not a trivial task because an efficient prodrug has to fulfil two prerequisites: (1) the Pt(IV)–ligand bond must be sufficiently stable so as not to dissociate prior to reaching the cancerous tissues; and (2) there must be an efficient activation pathway in the intracellular media of the malignant cells [12]. These two crucially important points have been assessed in a recent theoretical contribution by Ponte, Russo and Sicilia [10], who used density functional theory (DFT) calculations to simulate the mechanism for the reduction of asplatin. The reported theoretical outcomes allow us to understand the mechanism behind the reduction by mono-deprotonated ascorbic acid (AscH^–^), which is the predominant state of ascorbic acid at physiological pH [15].

We adapted this computational strategy to couple oxoplatin with other FDA-approved anti-cancer drugs. To this end, the aspirin moiety present in asplatin (marked in orange in Figure 1) was replaced by the set of molecules extracted from the AOD9 library (Figure 2). The resulting structures were then fully optimized. However, these geometries alone do not allow us to predict whether the Pt(IV)–ligand bond is sufficiently strong to prevent the early dissociation of the molecule, which is one of the main prerequisites for an efficient prodrug [12]. With the aim of comparing the stability of the bioactive ligands introduced into the axial position, we monitored the relative energy as the coordination distances were increased in steps of 0.1 Å.

## 3. Discussion

**Figure 3:**
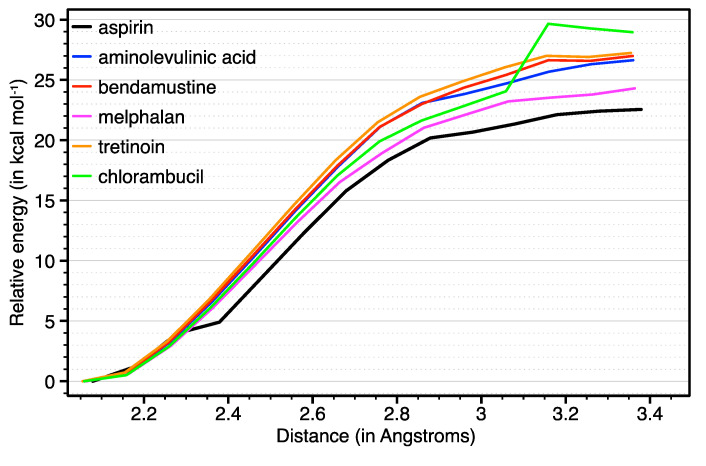
Relative energies (in kcal mol^–1^) that describe the dissociation of the bioactive ligands attached at the axial position of the Pt(IV) coordination sphere. The energy was monitored by increasing the Pt(IV)–ligand distance in steps of 0.1 Å, starting from the optimized geometry (ca. 2 Å).

The performed scans can be used to rank the novel prodrugs according to the lability of the attached bioactive ligand. Figure 3 shows that the relative energy associated with the detachment of the axial ligands increases with a very similar trend to that of aspirin (black line). A closer inspection reveals that aspirin is located ca. 23 kcal mol^–1^ above the computed energy for the coordinated moiety if the distance is increased to 3.4 Å. The proposed bioactive ligands lie within a range from 27 kcal mol^–1^ (melphalan, purple line) to 29 kcal mol^–1^ (chlorambucil, green line). For the latter, we observe a significant increase in the energy at ca. 3.1 Å. During that scan, the carboxylic group of the bioactive ligand is able to interact with the equatorial –NH_3_ groups through hydrogen-bonds (H-bonds). As a consequence, the combined breaking (Pt–O) and formation (NH_3_–O) of bonds might lead to complex energy curves such as the one displayed by chlorambucil. In spite of that specific dissimilarity in the computed curve, Figure 1 demonstrates that these ligands yield compounds as stable as aspirin (e.g., asplatin prodrug).

The Pt(IV) metallic centers are activated inside the cell as a result of the natural AscH^–^ gradient. The AscH^–^ reducing agent enters into the coordination sphere by forming a non-covalent interaction with the hydroxyl ligand at the axial position, the so-called inner electron transfer mechanism [16]. The structures in the preceding step are systematically re-optimized in the presence of AscH^–^. The constructed models correctly mimic the expected mechanism for a two-electron transference process, Pt(IV)→Pt(II). It has been shown that one of the hydrogen atoms in AscH^–^ is transferred to the hydroxyl ligand opposite the bioactive ligand in the initial stage [10]. This proton transfer subsequently activates the release of a water molecule that is concomitant with the detachment of the bioactive ligand at the opposite trans position. An analysis of the vibrational modes associated with each transition state confirmed that the reduction takes place at the same time as this mechanism. More specifically, we observe the transference of the proton towards the hydroxyl ligand while the single imaginary frequency corresponds to the reaction coordinate—that is, the departure of both ligands at the axial positions.

Table 1 lists the activation (ΔG^‡^) and reaction (ΔG) free energies for all possible activations. The corresponding Pt(IV)–AscH^–^ adduct is taken as the reference value for calculating the relative energies. The associated rate (k, s^–1^) and equilibrium (K_eq_) constants are also reported to further rationalize which prodrugs are the most prone to activation by ascorbic acid. We observed that most of the prodrugs underwent activation with an energetic barrier close to that of aspirin. The expected activation energies for aminolevulinic acid, aspirin, chlorambucil and melphalan differ by up to 1.87 kcal mol^–1^ only (ΔG^‡^ = 17.10–18.97 kcal mol^–1^), with rate constants of k ~10^−1^–10^–2^ s^–1^. Among all the assessed ligands, tretinoin is predicted to have the largest energetic barrier (ΔG^‡^ = 20.90 kcal mol^–1^) and is activated with a rate constant that is about one order of magnitude lower than for the other prodrugs (k ~10^–3^ s^–1^). Much larger differences are observed for the reaction free energies. The reduction of the prodrugs assembled with aminolevulinic acid and chlorambucil is expected to be similar to that of aspirin, with ΔG values in the range of –26.69 to –28.26 kcal mol^–1^. Consequently, a large negative value is predicted for the reduction if these drugs are used as the axial ligands, with an equilibrium completely shifted towards the activated drug (K_eq_ ~10^19^–10^20^). By contrast, the reduction of melphalan and, in particular, tretinoin occurs with a low efficiency, with equilibrium constants several orders smaller than those for the other prodrugs (K_eq_ ~10^3^–10^10^).

**Figure 4:**
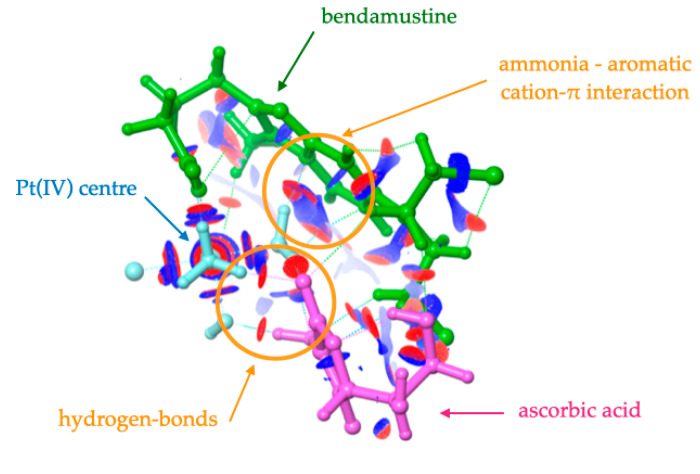
Optimized reactant for the prodrug formed by the Pt(IV) center (displayed in cyan) with bendamustine (in green) as the axial ligand, in the presence of ascorbic acid (plotted in purple). This figure also summarizes the non-covalent interaction analysis. Isosurfaces correspond to *s* = 0.5 au with a color scale of −0.01 au < ρ < 0.01 au, using density functional theory (DFT) densities. H-bonds are displayed as red spots, and weak interactions are represented as blue surfaces.

It should be stressed here that the simulation of the activation mechanism for bendamustine was unsuccessful. More specifically, the optimization of both the transition state and product quickly reverts to the initial prodrug, and consequently, it is not listed in Table 1. To correctly interpret this finding, we need to examine the optimized geometry and the electronic structure for the Pt(IV)–AscH^–^ adduct formed with that drug as the axial ligand. To this end, we computed the electron density and reduced density gradient (s) to visualize the noncovalent interactions through colored surfaces as described by Contreras-García, Yang and co-workers [17]. Figure 4 reveals a series of H-bonds and van der Waals contacts, displayed as red spots and blue surfaces, respectively. As expected, the interaction of the prodrug and the ascorbic acid is dominated by H-bonds established with the equatorial ammonia and the axial hydroxil ligands (circled red spots). We also observe several contacts between the ascorbic acid moiety and bendamustine. However, our attention should be focused on the middle region of the prodrug. As illustrated in Figure 4, a sizable surface appears between one of the ammonia ligands and the aromatic core of bendamustine, namely, the benzimidazole moiety. It is known that positively charged quarternary ammonium compounds (e.g., NH_4_^+^) are able to form a cation–π interaction with aromatic groups [18]. Similarly, the ammonia ligand is largely polarized when it enters into the coordination sphere of the Pt(IV). Indeed, the computed Mulliken charges for the hydrogen atoms in the isolated NH_3_ (*q* = 0.095|e|), NH_4_^+^ (*q* = 0.227|e|) and the NH_3_ ligand assembled in the prodrug with bendamustine (*q* = 0.213|e|) confirm the positive charge associated with the coordinate ammonia ligand. As illustrated in Figure 4, ammonia is therefore able to anchor the bendamustine ligand through a cation–π interaction. All these accumulated data hint that bendamustine is not the best precursor from which to assemble prodrugs due to that additional intra-ligand interaction, which in turn reduces the activation step.

**Figure 5:**
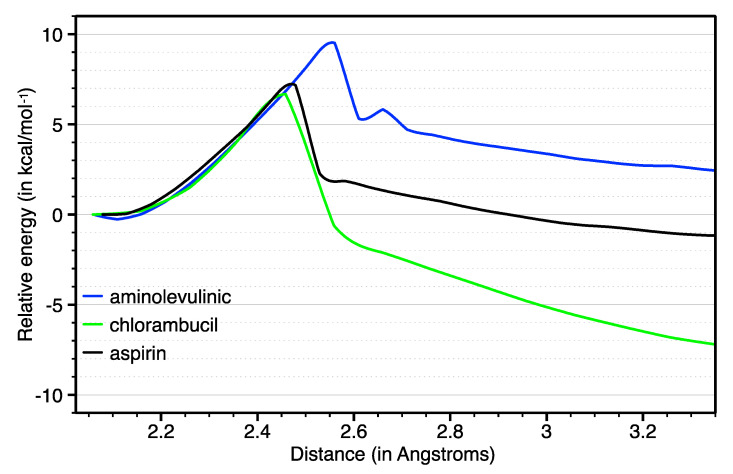
Relative energy (kcal mol^−1^) describing the dissociation of the bioactive ligands attached at the axial position for the one-electron reduced forms, e.g., with the Pt(III) metallic center. The energy was monitored by increasing the Pt(III)–ligand distance in steps of 0.05 Å, starting from the optimized geometry (ca. 2 Å). For the sake of clarity, we use the same color scheme as in Figure 3. The previous reported barrier for aspirin is 6.7 kcal mol^−1^. See [10].

There is one remaining crucial issue that needs to be addressed: the possible activation of the drug outside the target cell by a molecule other than ascorbic acid. Pt(IV)-based drugs are prone to activation by ascorbic acid with a second order rate constant of ~ 2 M^−1^ s^−1^ [19], which is present at high concentrations inside cells. It is worth noting that Pt(IV)-based products might be also reduced under physiological conditions by glutathione in the intracellular medium. However, experimental data suggest that the reduction of Pt(IV) prodrugs by glutathione leads to a cisplatin–glutathione adduct, which is not as toxic as free cisplatin to the cancer cell [20]. As far as the external medium is concerned, there are reducing species that might activate the prodrug before it reaches the cell, e.g., the thiol groups present in cysteine, thioglycolic acid and methionine [21]. The external activation might occur through an outer-sphere electron transfer mechanism, in which a six-coordinated Pt(III) intermediate is formed by the addition of a single electron before the prodrug reaches the cell [22]. Accordingly, we determined the non-selective activation in the extracellular medium by scanning the relative energy during the loss of the axial ligand upon one-electron reduction. Figure 5 shows the relative energies of the Pt(III) intermediates of the parent aspirin as well as the two best ranked prodrugs, e.g., those with aminolevulinic acid and chlorambucil as axial bioactive ligands (Table 1). As expected, one-electron reduction activates the release of the axial ligand in all cases, although the impact is not homogenous throughout the series of prodrugs. The one-electron reduced form of chlorambucil (green line) dissociates with a very similar energetic barrier to aspirin (black line), and their products are rapidly stabilized with the release of the axial ligand. However, the energetic profiles in Figure 5 suggest that aminolevulinic acid (blue line) might be used to increase the barrier.

The picture of the Pt(III) intermediate’s stability is completed by optimizing the reactants, transition states and products of the prodrug assembled with aspirin and the best ranked ligand, e.g., aminolevulinic acid. The numeric values for the activation and reaction energies are listed in Table 2. According to these calculations, the axial aspirin ligand in the one-electron reduced asplatin is lost at a cost of 5.60 kcal mol^−1^ (k ~10^–8^ s^–1^), while aminolevulinic acid increases the barrier to 8.41 kcal mol^−1^ (k ~10^–8^ s^–1^). The use of aminolevulinic acid also destabilizes the product of the dissociation by ca. 2 kcal mol^−1^ (K_eq_ = 5.18 × 10^–2^), which shifts the equilibrium towards the hexacoordinated Pt(III) form by two orders of magnitude compared to aspirin (K_eq_ = 1.46 × 10^0^). We should underline that these energies arise from the reactive adducts, the transition states and the product adducts and, consequently, could not be directly compared to other values computed by using separated reagents and products. However, the provided k and K_eq_ values in Table 2 provide meaningful insights in the impact of product stability upon axial ligand replacement.

The consensus of the simulations performed here suggests that aminolevulinic acid and chlorambucil are the best molecules for assembling oxoplatin-based prodrugs because (1) their coordination yields an inert compound that releases the axial bioactive ligand at least as slowly as aspirin; (2) according to the computed rate and equilibrium constants, they can be efficiently activated by ascorbic acid; and (3) they are better at preventing early release by reduction outside the target cell. Although requisites (1) and (2) are equally fulfilled by these two ligands, aminolevulinic acid outperforms all the other prodrugs as a result of the higher stability of the Pt(III) intermediate. To the best of our knowledge, there is no previous report of such a combination, although beneficial effects have been observed in combination with cisplatin. Terada and co-workers [23] have shown that aminolevulinic acid has a protective role during cisplatin-based chemotherapy because it reduces the nephrotoxicity of the treatment without interfering with the anti-cancer effects of cisplatin. In addition, aminolevulinic acid may be administered with cisplatin to increase the effectiveness of photodynamic therapies in cancer treatment because it acts as a photosensitizer for the accumulation of reactive oxygen species in malignant tissues [24,25,26,27]. In light of our results, the link to oxoplatin may be expanded to other porphyrin precursors used as photosensitizers, with the requisite of the presence of one carboxylic group [28].

## 4. Computational Methods

The asplatin geometry optimized by Ponte et al. [10] was used as a template to build our model systems. We replaced the aspirin moiety present in asplatin (marked in orange in Figure 1) with the anti-cancer drug set shown in Figure 2. The resulting structures were fully optimized at the B3LYP-D3 theory level, which consisted of Becke’s three-parameter hybrid exchange functional (B3); the correlation functional of Lee, Yang and Parr (LYP); and Grimme’s dispersion contribution term [29,30,31]. That approach has been shown to provide reliable results for the thermochemistry and kinetics of Pt-based compounds [32,33]. The def2-SVP basis set was used to describe all the atoms, except the Pt centers, where the core electrons were replaced with the effective core potentials def2-ECP to account for scalar relativistic effects without increasing the computational cost [34,35]. For all the calculations, we used an ultrafine grid for numerical density functional theory integration. The optimized structures were confirmed to be stable (real minima) or transitional state (saddle point) in the potential energy surface by analyzing their vibrational modes. No imaginary frequency was obtained for the products and reactants, although a single imaginary frequency was obtained for the transitional state. Pt(IV) and Pt(II) species correspond to singlet electronic configurations, while Pt(III) is a triplet state. Spin contamination for the latter was controlled during all the calculations (the maximum S^2^ value was only 0.7549). The free energies were calculated by adding the zero-point energy and thermal corrections at 298.15 K. Relative energy curves for the dissociation of axial ligands were computed by monitoring their distance from the oxygen atom bound to the metallic center. This is a relaxed potential energy scan over the reaction coordinate (i.e., the bond being dissociated). Because solvation might influence the thermodynamics and kinetics of the reactivity of Pt compounds [36], environmental effects were included by using the polarizable continuum method of Tomasi and co-workers [37]. All the optimizations were performed with Gaussian16 [38], while NCI analysis was conducted with the NCI analysis tool implemented in Jaguar 10.8 [39,40].

## 5. Conclusions

Despite increasing interest in the possible associations between oxoplatin and other drugs [41,42,43,44,45,46], there is currently no recommended approach to systematically improving the anti-cancer activity of bifunctional drugs based on Pt(IV). With the aim of contributing to the design of enhanced anti-cancer drugs, we used a state-of-the-art computational approach to screen the latest FDA-approved oncology drugs. Specifically, we searched for novel axial ligands to decorate the parent oxoplatin drug. The only restriction imposed was the presence of one carboxylic group in the structure, a chemical feature that enables coordination to the oxoplatin coordination sphere via a simple esterification procedure. Among all the designed molecules, the combination of oxoplatin with aminolevulinic acid and chlorambucil emerged as very promising oncology drugs because they fulfilled all the specific prerequisites: (1) the ligands were efficiently introduced into the Pt(IV) sphere of the coordination structure and therefore did not dissociate before entering the target cell; (2) the activation energy barrier was sufficiently low to allow rapid reduction by the ascorbic acid present in the intracellular medium; (3) the axial ligands were completely released when the Pt(IV) was reduced to Pt(II); and (4) the stability of the Pt(III) intermediate outside the target cell matched that of aspirin, used here as a reference. This stability was increased if aminolevulinic acid was used as the axial ligand. We recommend using theoretical tools to assess both the stability of the prodrug formed in neutral and anionic forms and its reactivity with ascorbic acid prior to the laboratory synthesis of any novel Pt(IV)-based compounds. We hope that these drug combinations and computational protocols will drive experimental efforts to synthesize enhanced Pt(IV) prodrugs.

## Figures and Tables

**Table 1:** Computed activation (ΔG^‡^) and changes in reaction (ΔG) free energy (kcal mol^−1^) for the reduction of the prodrugs by ascorbic acid and the detachment of the bioactive ligand. The associated rate (k, s^–1^) and equilibrium (K_eq_) constants are also given.

Axial Ligand	ΔG^‡^	ΔG	k	K_eq_
aminolevulinic acid	18.84	−26.69	9.42 × 10^−2^	3.80 × 10^19^
aspirin ^a^	18.41	−24.51	1.90 × 10^–1^	9.58 × 10^17^
chlorambucil	18.97	−28.26	7.56 × 10^–2^	5.36 × 10^20^
melphalan	17.10	−14.21	6.43 × 10^–1^	2.64 × 10^10^
tretinoin	20.90	−4.37	2.91 × 10^–3^	1.60 × 10^3^

[a] Previous reported energies for aspirin are ΔG^‡^ = 14.6 kcal mol^−1^ and ΔG = –24.7 kcal mol^−1^. See [10] for further details.

**Table 2:** Computed activation (ΔG^‡^) and changes in reaction (ΔG) free energy (kcal mol^−1^) with the detachment of the best ranked bioactive axial (aminolevulinic acid) compared to the reference values for aspirin in the one-electron reduced Pt(III) form. The associated rate (k, s^–1^) and equilibrium (K_eq_) constants are also given.

Axial Ligand	ΔG^‡^	ΔG	k	K_eq_
aminolevulinic acid	8.41	1.75	4.21 × 10^6^	5.18 × 10^–2^
aspirin ^a^	5.60	–0.22	4.83 × 10^8^	1.46 × 10^0^

[a] Previous reported activation energy for aspirin in the anionic form of the asplatin is ΔG^‡^ = 6.2 kcal mol^−1^. See [10] for further details.
